# Correlation of Hsp70 Serum Levels with Gross Tumor Volume and Composition of Lymphocyte Subpopulations in Patients with Squamous Cell and Adeno Non-Small Cell Lung Cancer

**DOI:** 10.3389/fimmu.2015.00556

**Published:** 2015-11-02

**Authors:** Sophie Gunther, Christian Ostheimer, Stefan Stangl, Hanno M. Specht, Petra Mozes, Moritz Jesinghaus, Dirk Vordermark, Stephanie E. Combs, Friedhelm Peltz, Max P. Jung, Gabriele Multhoff

**Affiliations:** ^1^Department of Radiation Oncology, Klinikum rechts der Isar, Technische Universität München (TUM), München, Germany; ^2^Department of Radiation Oncology, Martin Luther University Halle-Wittenberg, Halle (Saale), Germany; ^3^Department of Pathology, Klinikum rechts der Isar, Technische Universität München (TUM), München, Germany; ^4^Department of Innovative Radiotherapy (iRT), Helmholtz Zentrum München, Oberschleißheim, Germany; ^5^Department of Radiation Sciences (DRS), Helmholtz Zentrum München, Oberschleißheim, Germany; ^6^Pulmonary Division, 1 Medizinische Klinik, Klinikum rechts der Isar, Technische Universität München (TUM), München, Germany

**Keywords:** biomarker, tumor markers, biological, heat-shock protein 70, NSCLC, gross tumor volume, lymphocytes, immune responses

## Abstract

Heat-shock protein 70 (Hsp70) is frequently found on the plasma membrane of a large number of malignant tumors including non-small cell lung cancer (NSCLC) and gets released into the blood circulation in lipid vesicles. On the one hand, a membrane (m)Hsp70-positive phenotype correlates with a high aggressiveness of the tumor; on the other hand, mHsp70 serves as a target for natural killer (NK) cells that had been pre-stimulated with Hsp70-peptide TKD plus low-dose interleukin-2 (TKD/IL-2). Following activation, NK cells show an up-regulated expression of activatory C-type lectin receptors, such as CD94/NKG2C, NKG2D, and natural cytotoxicity receptors (NCRs; NKp44, NKp46, and NKp30) and thereby gain the capacity to kill mHsp70-positive tumor cells. With respect to these results, the efficacy of *ex vivo* TKD/IL-2 stimulated, autologous NK cells is currently tested in a proof-of-concept phase II clinical trial in patients with squamous cell NSCLC after radiochemotherapy (RCT) at the TUM. Inclusion criteria are histological proven, non-resectable NSCLC in stage IIIA/IIIB, clinical responses to RCT and a mHsp70-positive tumor phenotype. The mHsp70 status is determined in the serum of patients using the lipHsp70 ELISA test, which enables the quantification of liposomal and free Hsp70. Squamous cell and adeno NSCLC patients had significantly higher serum Hsp70 levels than healthy controls. A significant correlation of serum Hsp70 levels with the gross tumor volume was shown for adeno and squamous cell NSCLC. However, significantly elevated ratios of activated CD69^+^/CD94^+^ NK cells that are associated with low serum Hsp70 levels were observed only in patients with squamous cell lung cancer. These data might provide a first hint that squamous cell NSCLC is more immunogenic than adeno NSCLC.

## Introduction

According to recent statistics, lung cancer is still among the most frequent causes of cancer-related deaths and the second most common cancer in both men and women in Western societies (1). The numbers of new cases are further increasing especially in Asia and Africa (2). According to the GLOBOCAN report 2000 (3), the incidence of lung cancer worldwide is 1,238,900 with a mortality of 1,103,100 and a 5-year prevalence of 1,394,400. One reason for this high mortality is that patients with lung cancer are frequently diagnosed in advanced tumor stages since the symptoms, such as dyspnea, coughing, or chest pain, are quite unspecific for a long period of time (4). Even after radical surgery, chemo-, and/or radiotherapy using up-to-date therapeutic approaches could not improve the outcome of locally advanced tumor stages. The progression-free and overall survival of non-small cell lung cancer (NSCLC) patients in stage IIIA and IIB is often <16 months (5). Therefore, there is a high medical need to explore new treatment modalities to increase life expectancy and to develop minimal invasive methods for an earlier detection of NSCLC.

In 2013, immunotherapy was elected as the “breakthrough of the year” for the treatment of cancer by the journal “Science” (6). The basis for this was the increase in knowledge in the detection of tumor-specific traits that have the potential to serve as tumor-specific targets for immunotherapeutic approaches. Along this line, our laboratory investigated the potential of the major stress-inducible heat-shock protein 70 (Hsp70, HSPA1A) as a tumor-specific target. Hsp70 is frequently overexpressed in many different tumor types like hematological malignancies, breast, prostate, colon, brain, and lung cancer (7, 8). Hsp70 assists protein folding, prevents protein aggregation and apoptotic cell death under physiological conditions and following stress (9, 10). Tumor cells compared to normal cells not only express significantly higher levels of Hsp70 in the cytosol (7, 8), but also exhibit an unusual plasma membrane localization of Hsp70 (11). Therefore, mHsp70 has the potential as a tumor-specific target for immunological approaches. Additionally, we have shown recently that mHsp70 positive tumor cells actively secrete Hsp70 in lipid vesicles, most likely exosomes, that mirror the membrane orientation of the cell from which they are derived (12). Based on these findings, liposomal Hsp70 which is found in the peripheral blood circulation can reflect the mHsp70 status of the tumor. We have established the lipHsp70 ELISA (13), which enables the detection of Hsp70 in the serum and plasma of patients. The use of the monoclonal antibody (mAb) cmHsp70.1 (14, 15) in this ELISA allows a quantitative determination of free and liposomal Hsp70 in the blood, whereas other commercially available Hsp70 ELISA tests only detect free Hsp70.

A mHsp70-positive tumor phenotype exerts dual functions, on the one hand, a high mHsp70 density is associated with a high aggressiveness of the tumor (16) and the potential of metastatic spread; on the other hand, mHsp70 on tumor cells serves as a target for activated natural killer (NK) cells, which have been incubated either with Hsp70 protein or TKD a 14mer peptide derived from Hsp70 in combination with low-dose IL-2 (TKD/IL-2) (12, 17, 18). Following activation, these NK cells regain the capacity to kill mHsp70-positive tumor cells *in vitro* (19) and *in vivo* (15, 20) via granzyme B-mediated apoptosis (21).

For a better understanding of this duality of mHsp70, we addressed the question whether serum Hsp70 levels are associated with clinical parameters, such as gross tumor volume (GTV) at diagnosis and after radiochemotherapy (RCT), and whether serum Hsp70 levels can have impact on the immune phenotype of squamous cell and adeno NSCLC (18).

## Materials and Methods

### Patient Material

Blood samples of healthy human donors and NSCLC patients of the Klinikum rechts der Isar, TUM (patient collective #1; Table 1) and the Martin Luther University Hospital Halle-Wittenberg (patient collective #2, Table 2) were collected between 2008 and 2015. In patient collective #1, blood was taken from patients with squamous cell (*n* = 25) and adenocarcinoma (*n* = 18) of the lung at diagnosis and directly after RCT (*n* = 6), and from age- and gender-matched healthy human volunteers (*n* = 126) as a control group. Tumor biopsies were obtained from nine NSCLC patients, six in stage IV, and three in stage 3 (patient collective #1). The median age of all patients of patient collective #1 was 64 years and ranged from 23 to 95 years. In a screening study, NSCLC patients are stratified for their tumor stage and Hsp70 phenotype to enter a phase II clinical trial at the TUM, which is entitled “Targeted NK cell based adoptive immunotherapy for the treatment of patients with NSCLC after radiochemotherapy (RCT)” (18). In patient collective #2, blood was taken from 55 patients (median age 63 years, range 47–86 years) with advanced stage, inoperable NSCLC with an indication for primary RCT. These patients were recruited into a pilot study entitled “Potential plasma hypoxia markers in the radiotherapy of non-small cell lung cancer” (22). Characteristics of both patient collectives are summarized in Tables 1 and 2. Briefly, blood was collected in two EDTA KE/9 ml tubes and one Serum Z/9 ml separator tube (S-Monovette, Sarstedt, Nümbrecht, Germany). For the serum, blood was allowed to clot for 15 min at room temperature. After collecting 1.4 ml of EDTA blood for flow cytometry, plasma and serum were obtained by centrifugation at 750 *g* for 10 min. Aliquots of 100–300 μl were stored at −80°C for further analysis. The studies were approved by the local Ethics Committee of the Medical Faculties of both Universities (TUM, Halle-Wittenberg) and written informed consent was obtained from all patients before entering the trial. All procedures were performed in accordance to the Declaration of Helsinki, 1975, as revised in 2008. Clinical stage was determined according to the UICC TNM classification, seventh edition.

**Table 1:** **Patient collective #1**.

		Number
Gender	Female	11
	Male	32
Histology	Squamous cell	25
	Adeno	18
UICC stage	Ia	1
	Ib	1
	IIa	0
	IIb	0
	IIIa	13
	IIIb	13
	IV	15

*Clinico-pathological characteristics of 43 NSCLC patients treated at the Klinikum rechts der Isar, TU München, Munich, Germany*.

**Table 2:** **Patient collective #2**.

		Number
Gender	Female	8
	Male	47
Histology	Squamous cell	28
	Adeno	24
	Large cell	1
	Other	1
	No histology	1
UICC stage	Ia	1
	Ib	0
	IIa	2
	IIb	0
	IIIa	18
	IIIb	34
	IV	0

*Clinico-pathological characteristics of 55 NSCLC patients treated at the Martin-Luther University Hospital, Halle-Wittenberg*.

### Radiochemotherapy and Volumetric Parameters

Three-dimensional conformal RT (3D-RT) was given normofractionated (5 fractions/week) with curative intent (66 Gy total dose, 2 Gy single dose; Siemens Primus, Germany). Chemotherapy consisted of cisplatin (20 mg/m^2^ body surface on days 1–5) and vinorelbine (25 mg/m^2^ body surface on day 1) in treatment week 1 and 5 (2 courses). RT was CT based (Siemens Lightspeed RT, Germany) and all patients received a PET-scan (Philips Accel, USA) before RT. CT and PET images were merged and GTV was defined as the primary tumor and involved nodes (pathologic confirmed, highly suspicious by CT and PET). GTV was delineated by an experienced radiation oncologist at planning CT before RT and all image data were registered in the Oncentra Masterplan external beam planning software (Nucletron, USA) used for RT plan calculation.

### Detection of Hsp70 in Serum/Plasma Using the lipHsp70 ELISA

The Hsp70 content in the blood of NSCLC patients and healthy donors was determined using the lipHsp70 ELISA, which is equally suitable for serum and plasma samples (13). Using the monoclonal cmHsp70.1 antibody as a detection reagent (15), it is possible to detect both, soluble-free and lipid-bound Hsp70 in the serum/plasma of patients and healthy human individuals. This ELISA allows a quantitative analysis of the total amount of Hsp70 in the circulation blood (13). Briefly, 96-well MaxiSorp Nunc-Immuno plates (Thermo, Rochester, NY, USA) were coated overnight with 2 μg/ml rabbit polyclonal antibody (Davids, Biotechnologie, Regensburg, Germany), directed against human Hsp70 in sodium carbonate buffer (0.1 M sodium carbonate, 0.1 M sodium hydrogen carbonate, pH 9.6). After washing three times with phosphate-buffered saline (PBS, Life Technologies, Carlsbad, CA, USA) with 0.05% Tween-20 (Calbiochem, Merck, Darmstadt, Germany), wells were blocked with 2% milk powder (Carl Roth, Karlsruhe, Germany) in PBS for 1.5 h at 27°C. Following another washing step, serum samples diluted 1:5 in CrossDown Buffer (AppliChem, Chicago, IL, USA) were added to the wells for 2 h at 27°C. Then, the wells were washed again and incubated with 4 μg/ml of the biotinylated mouse mAb cmHsp70.1 (multimmune, Munich, Germany) in 2% milk powder in PBS for 2 h at 27°C. Finally, after another washing step, 0.2 μg/ml horseradish peroxidase-conjugated streptavidin (Pierce, Thermo, Rockford, IL, USA) in 1% bovine serum albumin (Sigma-Aldrich, St. Louis, MO, USA) was added for 1 h at 27°C. Binding was quantified by adding substrate reagent (R&D Systems, Minneapolis, MN, USA) for 30 min at 27°C and absorbance was read at 450 nm, corrected by absorbance at 570 nm, in a Microplate Reader (BioTek, Winooski, VT, USA). An eight-point standard curve was determined for each ELISA test using 0–50 ng/ml recombinant Hsp70 diluted in CrossDown Buffer. Each sample was measured in triplicates.

### Immunohistochemical Staining

Immunohistochemical staining was performed on formalin-fixed and paraffin-embedded specimen of lung tumors (*n* = 9). Sections were cut, dewaxed and hydrated, heated for 30 min in a microwave oven in 600 ml DAKO retrieval buffer, then washed for 5 min in H_2_O. After washing twice with T-PBS buffer, specimens were blocked for 1 h in 10% rabbit serum in PBS containing 1% BSA. Immunohistochemistry was done with streptavidine–biotin complex (StreptABC) using mouse mAb cmHsp70.1 (multimmune, Munich Germany) at a dilution of 1:200 overnight 2 h at 4°C.

### Analysis of the Lymphocyte Subpopulations with Flow Cytometry

In order to determine the proportion of different lymphocyte subpopulations, flow cytometric (FACS) analysis was performed using freshly collected EDTA blood (1.4 ml). Therefore, blood (100 μl) was transferred into 14 test tubes and then fluorescently labeled antibodies were added. The antibody combinations that were used for the FACS analysis are summarized in Table 3. After an incubation time of 15 min in the dark, the tubes were centrifuged for 5 min at 500 *g* at room temperature after adding 2 ml of PBS/10% FCS washing buffer. In order to eliminate erythrocytes, cells were incubated with lysing buffer (1:9 dilution of BD Lysing Solution Cat. 3490202 with millipore H_2_O) for 10 min at the room temperature in the dark. The respective percentages of B, T, and NK cell subpopulations are defined as the proportion of cells within the lymphocyte gate R1 (see Figure 3). For the determination of regulatory T cells, buffer A (1:10 dilution of component A with H_2_O) was added to the respective tubes. After two washing steps, cells were permeabilized with buffer C (1:50 dilution of buffer A with component B) for 30 min in the dark. Following another two washing steps, a PE-conjugated antibody directed against the intracellular transcription factor forkhead box P3 (FoxP3) was added for another 30 min. After another two washing steps, 5 × 10^4^ cells were analyzed on a FACScalibur instrument (Becton Dickinson, Heidelberg, Germany).

**Table 3:** **Panel of antibodies and 14 antibody combinations used in the study**.

Specificity	Antibody	Clone	Company	Cat No.	Volume
Ctrl	IgG1-FITC	X40	BD	345815	5
	IgG1-PE	X40	BD	345816	5
	IgG1-PerCP	X40	BD	345817	5
	IgG1-APC	X40	Caltag/Invitrogen	MG 105	1
T/NK	CD94-FITC	HP-3D9	BD	555888	5
	CD56-PE	NCAM16.2	BD	345811	5
	CD3-PerCP	SK7	BD	345766	10
	CD45-APC	HI30	Caltag/Invitrogen	MHCD 4505	1
B/T/NK	CD56-FITC	NCAM16.2	BD	345811	5
	CD19-PE	HIB19	BD	555413	20
	CD3-PerCP	SK7	BD	345766	10
	CD45-APC	HI30	Caltag/Invitrogen	MHCD 4505	1
T/NK	CD56-FITC	NCAM16.2	BD	345811	5
	CD16-PE	3G8	BD	555407	10
	CD3-PerCP	SK7	BD	345766	10
	CD45-APC	HI30	Caltag/Invitrogen	MHCD 4505	1
T/NK	CD56-FITC	NCAM16.2	BD	555518	5
	NKG2D-PE	149810	R&D	FAB139P	10
	CD3-PerCP	SK7	BD	345766	10
	CD69-APC	L78	BD	340560	5
T/NK	CD56-FITC	NCAM16.2	BD	345811	5
	NKp30-PE	IM3709	BC	PN 3709	10
	CD3-PerCP	SK7	BD	345766	10
	CD69-APC	L78	BD	340560	5
T/NK	CD56-FITC	NCAM16.2	BD	345811	5
	NKp46-PE	IM3711	BC	PN 3711	10
	CD3-PerCP	SK7	BD	345766	10
	CD69-APC	L78	BD	340560	5
T/NK	CD94-FITC	HP-3D9	BD	555888	5
	NKG2D-PE	149810	R&D	FAB139P	10
	CD3-PerCP	SK7	BD	345766	10
	CD56-APC	B159	BD	555518	10
T/NK	CD94-FITC	HP-3D9	BD	555888	5
	NKp30-PE	IM3709	BC	PN 3709	10
	CD3-PerCP	SK7	BD	345766	10
	CD56-APC	B159	BD	555518	10
T/NK	CD94-FITC	HP-3D9	BD	555888	5
	NKp46-PE	IM3711	BC	PN 3711	10
	CD3-PerCP	SK7	BD	345766	10
	CD56-APC	B159	BD	555518	10
CD4/CD8 T	CD4-FITC	RPA-T4	BD	555346	20
	CD8-PE	RPA-T8	BD	555366	20
	CD3-PerCP	SK7	BD	345766	10
	CD45-APC	HI30	Caltag/Invitrogen	MHCD 4505	1
Ctrl	IgG1-FITC	X40	BD	345815	5
	IgG1-PE	X40	BD	345816	5
	IgG1-PerCP	X40	BD	345817	5
	IgG1-APC	X40	Caltag/Invitrogen	MG 105	1
CD4 Treg	CD4-PE	RPA-T4	BD	555346	20
	FoxP3-FITC	259/C7	BD	560046	20
	CD3-PerCP	SK7	BD	345766	10
	CD25-APC	2A3	BD	340907	5
CD8 Treg	CD8-PE	RPA-T8	BD	555366	20
	FoxP3-FITC	259/C7	BD	560046	20
	CD3-PerCP	SK7	BD	345766	10
	CD25-APC	2A3	BD	340907	5

*APC, allophycocyanin; B, B lymphocyte; BD, Becton Dickinson Biosciences; BC, Beckman Coulter; CD, cluster of differentiation; COPD, chronic obstructive pulmonary disease; Ctrl, control; FITC, fluorescein isothiocyanate; NK, natural killer cell; PE, phycoerythrin; T, T lymphocyte; Treg, regulatory T cells*.

### Statistical Analysis

Statistical analysis was performed using the IBM SPSS 20.0 software package for windows (SPSS Inc., USA). Statistically significant differences between Hsp70 levels of patients with high and low GTV and high and low CD94 expression, lymphocyte subpopulations of healthy donors, patients with squamous cell and adenocarcinoma as well as between the percentage of all lymphocytes of patients with high and low Hsp70 expression were determined with Mann–Whitney’s *U*-test. Correlation between serum Hsp70 levels and GTV was evaluated using Spearman’s Rank Correlation Coefficient. Potential differences in Hsp70 serum levels in NSCLC patients before and after RCT were determined with the Wilcoxon Rank-Sum Test. Comparison of Hsp70 levels in the serum of two patient groups (squamous cell and adeno NSCLC) and a group of healthy donors was also performed using the Kruskal–Wallis test with a Dunn multiple comparison test. A value of *p* < 0.05 was considered as statistically significant.

## Results

### Comparison of Hsp70 Levels in Patients with Squamous Cell, Adeno NSCLC, and Healthy Human Individuals

**Figure 1:**
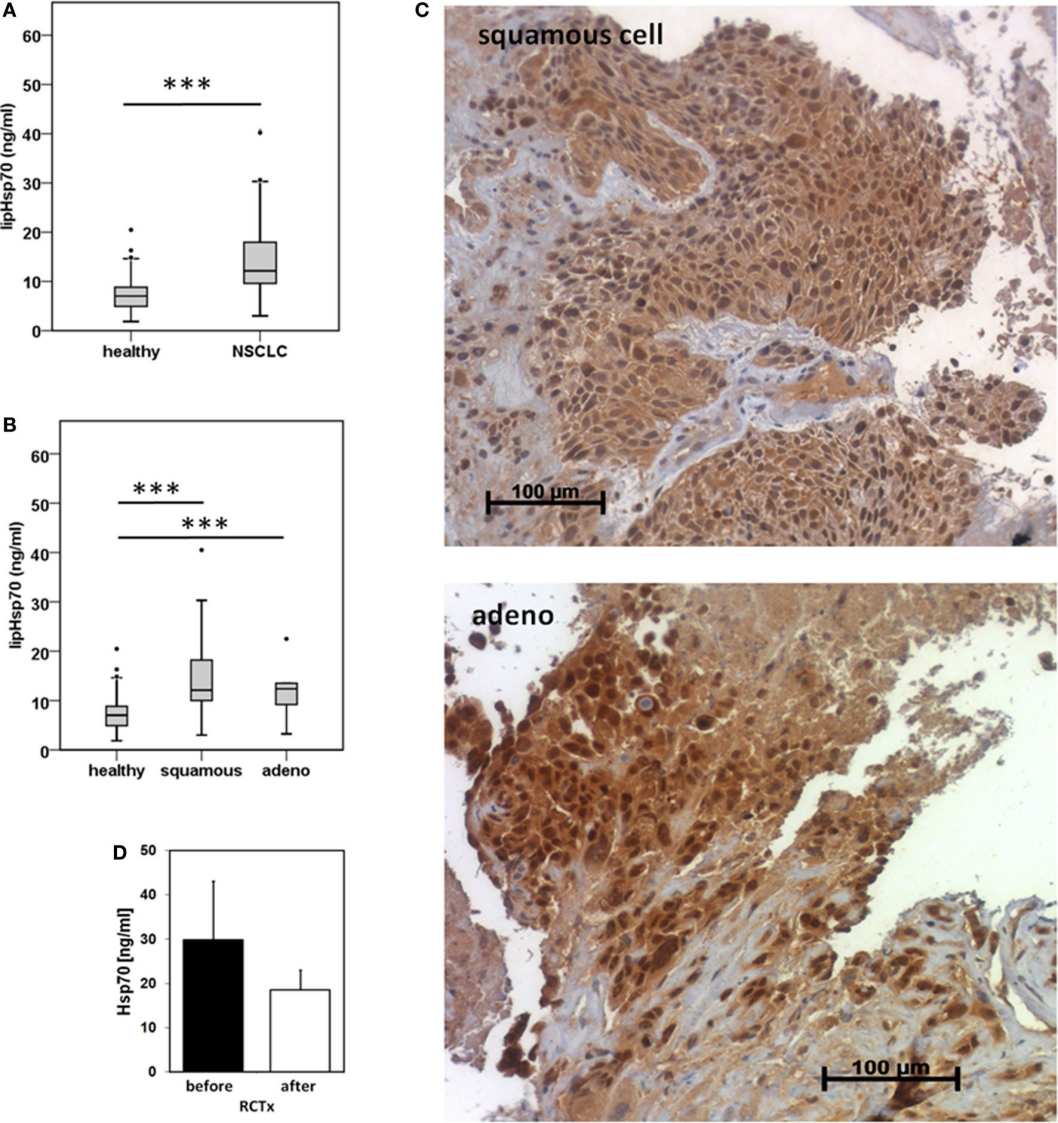
**Hsp70 serum levels (nanogram per milliliter) in healthy human individuals and patients with squamous cell and adeno NSCLC at diagnosis and directly after RCT therapy**. **(A)** Serum Hsp70 levels of healthy donors (healthy; *n* = 126; median Hsp70 level: 7.03 ng/ml, 95th percentile: 13.84 ng/ml) and NSCLC patients (NSCLC; *n* = 43; median Hsp70 level: 12.15 ng/ml, 95th percentile = 40.20 ng/ml) (patient collective #1) at diagnosis measured with the lipHsp70 ELISA; ***p < 0.001 (Mann–Whitney U-Test). **(B)** Serum Hsp70 levels of healthy donors (healthy; *n* = 126) and patients with squamous cell (squamous; *n* = 25; median Hsp70 level: 12.10 ng/ml; 95th percentile: 40.50 ng/ml) and adeno (adeno; *n* = 18; median Hsp70 level: 12.38 ng/ml; 95th percentile: 40.20 ng/ml) NSCLC (patient collective #1) at diagnosis measured with the lipHsp70 ELISA; ***p < 0.001 (Kruskal–Wallis test with Dunn’s multiple comparison test). **(C)** Representative immunohistochemical images of a squamous cell and adeno NSCLC section stained with cmHsp70.1 antibody; 20× magnification (patient collective #1). The upper graph shows a squamous cell NSCLC and the lower graph an adeno NSCLC section. Only the tumor tissue but not the surrounding tissue shows an Hsp70 staining. **(D)** Serum Hsp70 levels of NSCLC patients (*n* = 6; patient collective #1) at diagnosis (before) and directly after RCT (after).

Serum samples derived from 25 squamous cell and 18 adeno NSCLC patients (patient collective #1), and 126 age- and gender-matched healthy donors were analyzed to determine the Hsp70 levels in the peripheral blood. As shown in Figure 1A, patients with squamous cell and adeno NSCLC (NSCLC; *n* = 43) have significantly higher serum Hsp70 levels (*p* ≤ 0.001) compared to healthy control controls (healthy; *n* = 126), as determined with the lipHsp70 ELISA, at diagnosis. The mean serum Hsp70 levels of patients with squamous cell and adeno NSCLC, 16.69 ± 2.7 ng/ml and 14.51 ± 2.49 (median 12.15 vs. 12.20 ng/ml), respectively, did not differ significantly from each other (*p* = 0.825), but both tumor types differed significantly (*p* ≤ 0.001) from that of healthy controls (7.0 ng/ml) as shown in Figure 1B. A representative image of an Hsp70 positive tumor section of a squamous cell (upper graph) and an adeno (lower graph) NSCLC in stage IV is illustrated in Figure 1C. All nine tumor sections of NSCLC patients (six in stage IV and three in stage III) had elevated Hsp70 serum levels and exhibited a strong Hsp70 staining in the tumor cells but not in the connective tissue. Studies are ongoing that aim to analyze a potential correlation between cytosolic and serum Hsp70 levels in a larger panel of patients.

To investigate whether RCT impacts on serum Hsp70 levels in a small subgroup of patients (*n* = 6), blood was collected before start of therapy and directly after completion of therapy. Although Hsp70 levels after RCT remained significantly higher compared to those of healthy human individuals, a slight drop, which did not reach statistical significance (*p* = 0.463; Wilcoxon Rank-Sum Test), was detectable in the serum Hsp70 levels after completion of RCT (Figure 1D).

### Correlation of Hsp70 Levels at Diagnosis with Tumor Volume in NSCLC Patients

**Figure 2:**
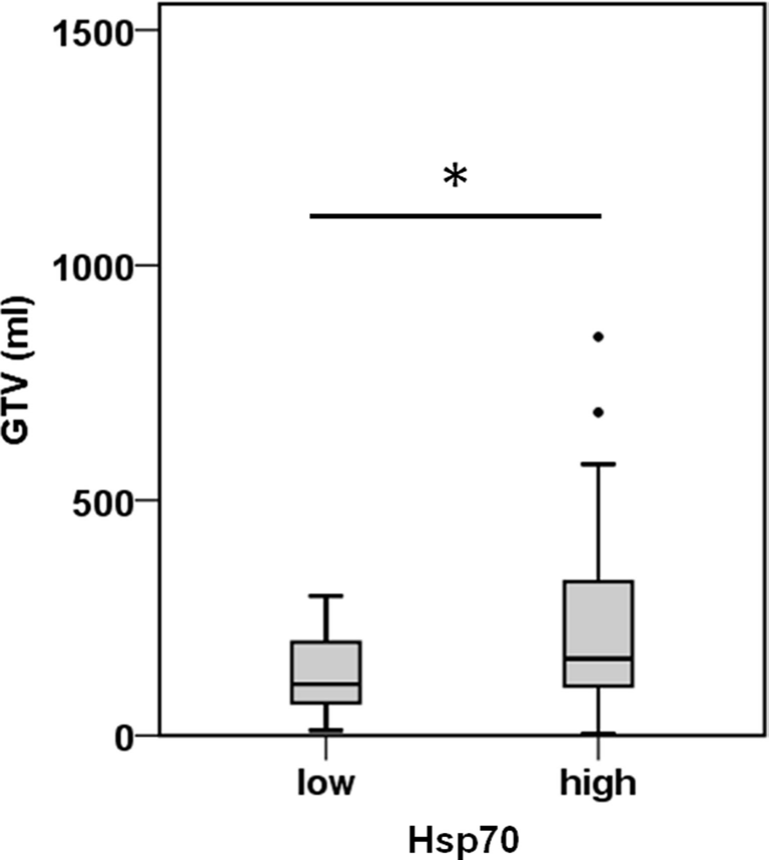
**Hsp70 serum levels and gross tumor volume (GTV) in NSCLC patients**. According to their median Hsp70 levels, NSCLC patients (*n* = 55; patient collective #2) were divided into patients with low GTV (≤143.6 ml; median GTV: 109.40 ml; 95th percentile: 296.30 ml) and high GTV (>143.6 ml; median GTV: 163.50 ml; 95th percentile: 688.00 ml); *p < 0.05 (Mann–Whitney *U*-test).

A comparison of free and lipid-bound Hsp70 in the circulation of tumor patients revealed that a major part of Hsp70 is bound to lipid vesicles, most likely exosomes, which are actively secreted by viable tumor cells carrying Hsp70 on their cell surface (12, 23). Therefore, we studied a potential correlation of the detected serum Hsp70 levels with the GTV of 55 NSCLC patients (patient collective #2; Table 2) that was determined by PET-imaging before start of RCT. The average tumor size of these patients was 219.9 ± 32.3 ml and the mean Hsp70 level was 11.2 ± 1.7 ng/ml. The Spearman’s Rank Correlation Coefficient revealed a significant correlation (*p* = 0.03) between these two metric parameters. Regarding the median GTV of 143.6 ml, these patients were subdivided into a group with low (≤143.6 ml) and high (>143.6 ml) median GTV. As shown in Figure 2, patients in the high GTV group had significantly higher serum Hsp70 levels than patients with a low tumor volume (*p* < 0.05).

### Differences in the Immune Phenotype of Healthy Human Donors, Patients with Squamous Cell and Adenocarcinoma of the Lung

**Figure 3:**
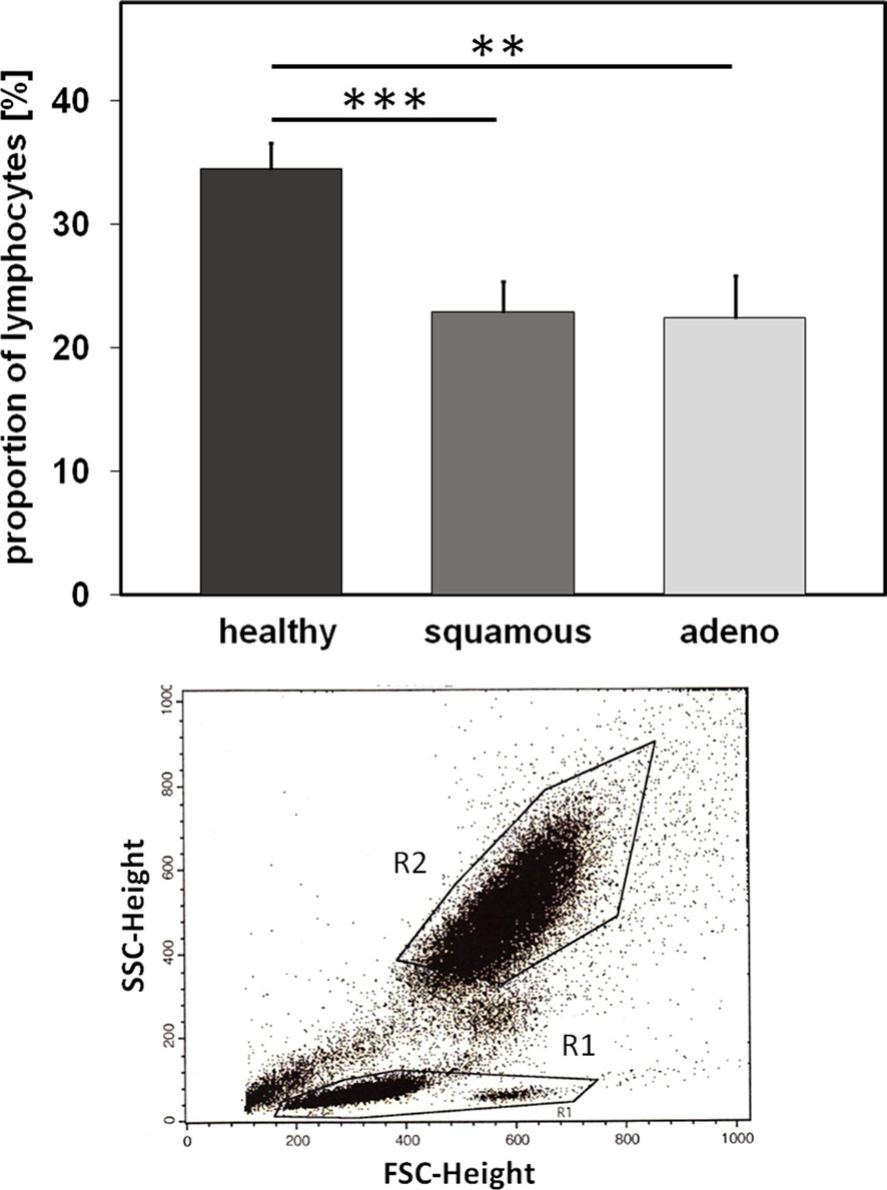
**Relative amounts of lymphocytes (%) in healthy human individuals and patients with squamous cell and adeno NSCLC**. Comparison of the percentage of peripheral blood lymphocytes (PBL) in healthy human individuals (*n* = 10) and patients with squamous cell (*n* = 25) and adeno (*n* = 18) NSCLC at diagnosis (patient collective #1); **p < 0.01, ***p < 0.001 (Mann–Whitney U-test). Graph below: gate R1 refers to the population of PBL which is analyzed by FACS, G2 refers to the population of granulocytes.

Nowadays, it is well accepted that an intact immune system plays a key role in long-term tumor control and in prevention of distant metastasis (24). Therefore, we comparatively investigated differences in the relative amount of lymphocytes and lymphocyte subpopulations, such as B, T, and NK cells, in the EDTA blood of healthy human donors (*n* = 10) and NSCLC patients (patient collective #1; *n* = 43; Table 1) at diagnosis, using multi-color FACS analysis. The panel of fluorescence-labeled antibodies and antibody combinations that were used in the study, is summarized in Table 3. Compared to blood of healthy human donors, the relative number of lymphocytes was significantly lower in patients with squamous cell (*n* = 25; *p* = 0.001) and adeno (*n* = 18; *p* = 0.008) NSCLC, although the percentage of lymphocytes in patients with different histology was very similar (22.4 ± 1.6% for squamous cell and 21.8 ± 2.4% for adenocarcinoma patients vs. 34.5 ± 1.77% in healthy controls) (Figure 3). The gating strategy of the lymphocytes is exemplified in the lower part of Figure 3. Gate R1 refers to the gated population of lymphocytes, whereas gate R2 represents granulocytes. The population of CD14^+^ monocytes is localized between the gates R1 and R2.

**Figure 4:**
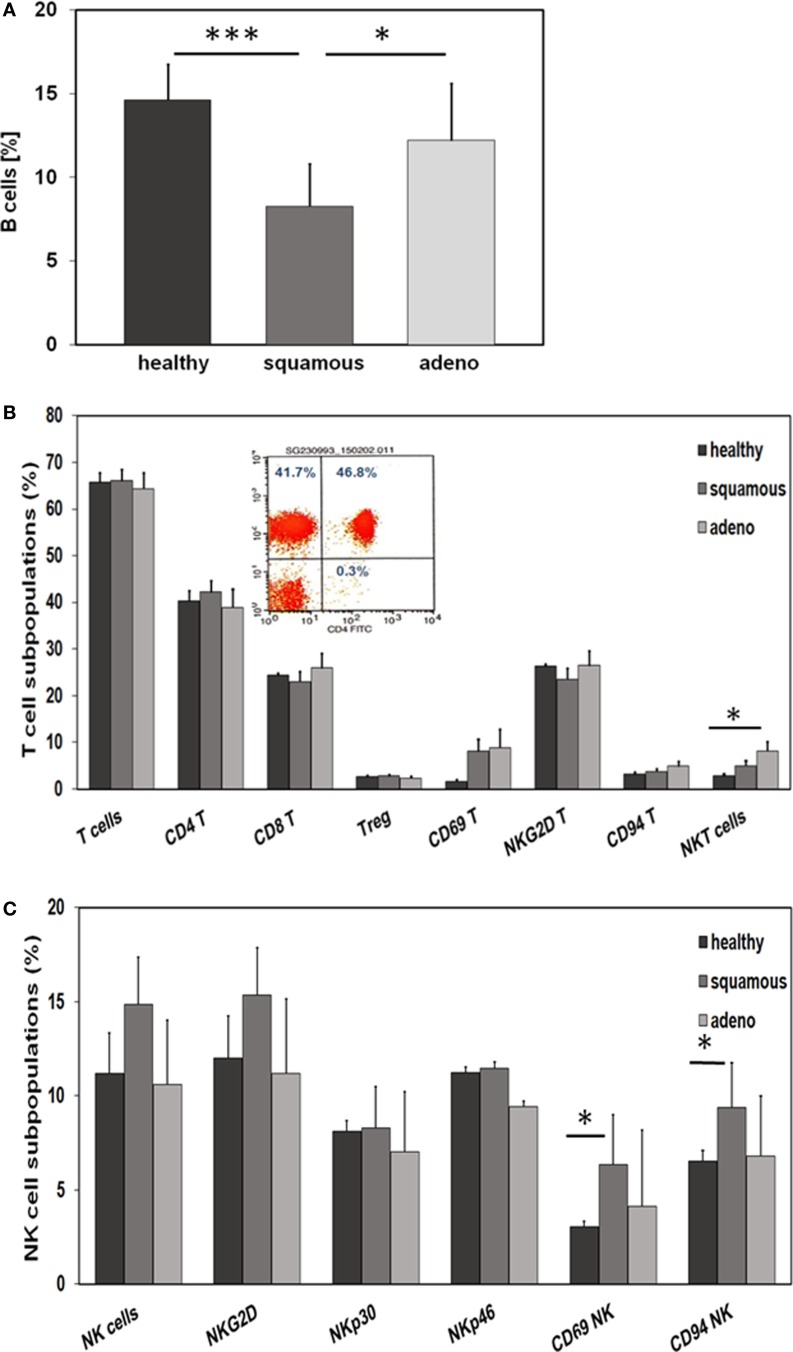
**Comparison of the immune phenotype of squamous cell and adeno NSCLC patients with healthy donors**. **(A)** Relative amount of B cells in the blood of healthy donors (*n* = 10), squamous cell (*n* = 25) and adeno (*n* = 18) NSCLC patients, as determined by FACS analysis (patient collective #1); *p < 0.05, ***p = 0.001 (Mann–Whitney *U*-test). **(B)** Percentage of T cell subpopulations in the blood of healthy donors (*n* = 10), squamous cell (*n* = 25) and adeno (*n* = 18) NSCLC patients (patient collective #1); *p < 0.05 (Mann–Whitney *U*-test). A representative example of the analysis of CD3^+^/CD4^+^ helper T cells by FACS analysis is shown in the inset (percentages of positively stained cells are shown in each quadrant of the dot blot). **(C)** Percentage of NK cell subpopulations in the blood of healthy donors (*n* = 10), squamous cell (*n* = 25) and adeno (*n* = 18) NSCLC patients (patient collective #1); *p < 0.05 (Mann–Whitney *U*-test).

With respect to CD19^+^ B cells, patients with squamous cell carcinoma had significantly lower percentages of B cells than healthy donors (*p* = 0.001) and adenocarcinoma patients (*p* = 0.02) (Figure 4A). A comparison of different CD3^+^ T cell subpopulations, such as CD4^+^, CD8^+^, CD4^+^/CD25^+^ regulatory, NKG2D^+^, and CD94^+^ T cells revealed no major differences, apart from a significant increase in the subpopulation of CD3^+^/CD56^+^ NKT cells in patients with adeno lung carcinomas compared to healthy controls (*p* = 0.038) (Figure 4B). The activation marker CD69 appeared to be slightly, but not significantly, elevated on CD3^+^ T cells of patients with squamous cell (*p* = 0.81) and adenocarcinoma (*p* = 0.197) patients compared to healthy controls (Figure 4B). A representative picture of the strategy to analyze CD3^+^/CD4^+^ T cells is illustrated in the inset of Figure 4B. In contrast to the T cell subpopulations, significant differences were observed with respect to CD3^−^ NK cell subpopulations regarding the activation marker CD69 and the C-type lectin receptor CD94. Patients with squamous cell NSCLC had significantly higher percentages of CD3^−^/CD56^+^/CD69^+^ (*p* = 0.016) and CD3^−^/CD56^+^/CD94^+^ (*p* = 0.028) NK cells than healthy controls (Figure 4C). Although not significantly different, it also appeared that patients with squamous cell NSCLC had elevated ratios of CD3^−^/CD56^+^ NK cells and CD3^−^/NKG2D^+^ NK cells in general, compared to healthy controls (*p* = 0.053) and adenocarcinoma patients (Figure 4C).

### Correlation of Hsp70 Serum Levels with Lymphocyte Subpopulations in Squamous Cell and Adenocarcinoma Patients

**Figure 5:**
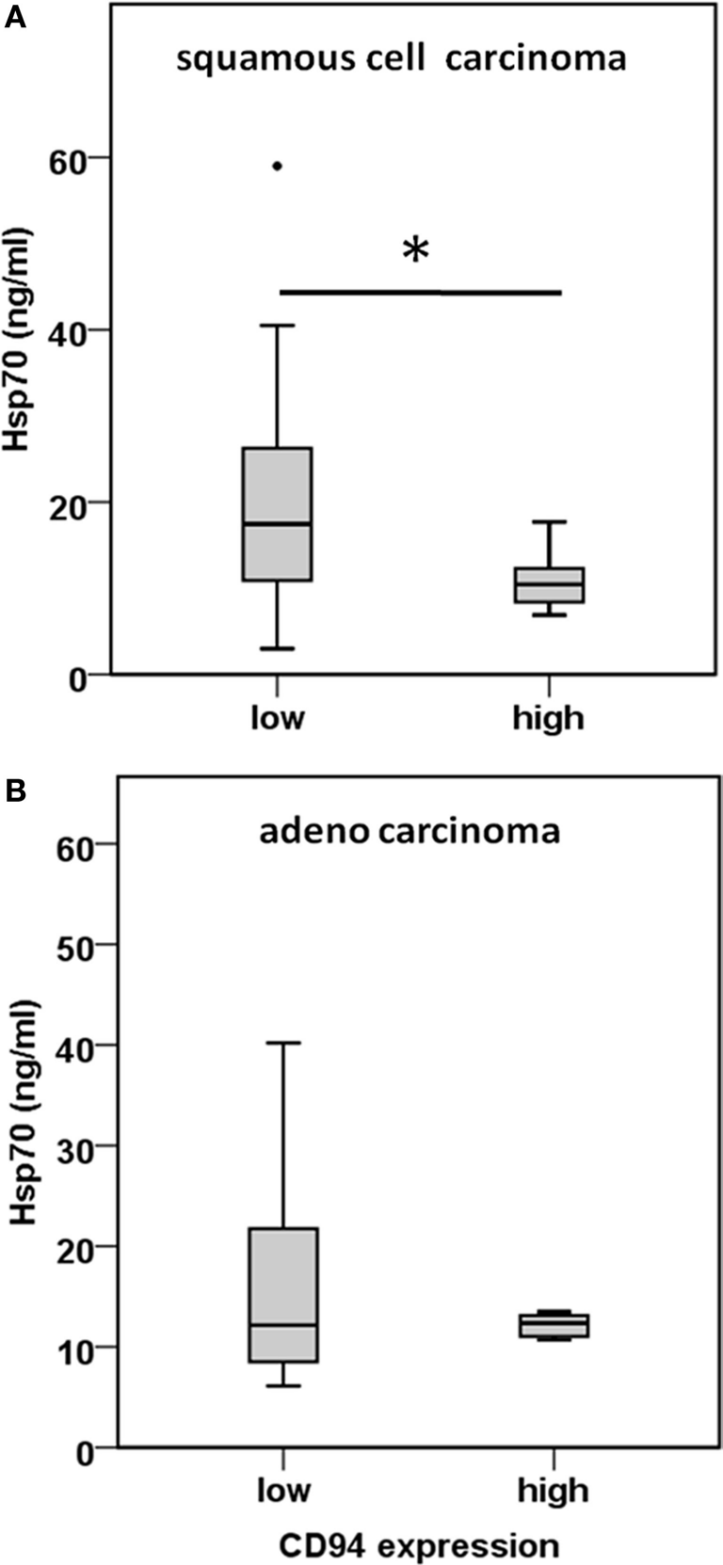
**Comparison of the CD94 immune phenotype and Hsp70 serum levels in squamous cell and adeno NSCLC patients**. **(A)** Correlation of the CD94 expression and serum Hsp70 levels in squamous cell NSCLC patients (*n* = 25) (patient collective #1). NSCLC patients were divided in a group with low (≤8.5% CD3^−^/CD94^+^ NK cells; median Hsp70 level: 17.45 ng/ml; 95th percentile: 59.00 ng/ml) and high (>8.5% CD3^−^/CD94^+^ NK cells; median Hsp70 level: 10.45 ng/ml; 95th percentile: 17.70 ng/ml) percentage of CD3^−^/CD94^+^ NK cells; *p < 0.05 (Mann–Whitney *U*-test). **(B)** Correlation of the CD94 expression and serum Hsp70 levels in adeno NSCLC patients (*n* = 18) (patient collective #1). NSCLC patients were divided in a group with low (≤6.5% CD3^−^/CD94^+^ NK cells; median Hsp70 level: 12.15 ng/ml; 95th percentile: 40.20 ng/ml) and high (>6.5% CD3^−^/CD94^+^ NK cells; median Hsp70 level: 12.35 ng/ml; 95th percentile: 22.50 ng/ml) percentage of CD3^−^/CD94^+^ NK cells; *p < 0.05 (Mann–Whitney *U*-test).

CD3^−^/CD94^+^ NK cells were found to be significantly elevated in squamous cell carcinoma of the lung (Figure 4C). Furthermore, we have shown earlier that the C-type lectin receptor CD94 plays a key role in the recognition of Hsp70 by NK cells (17). To investigate the influence of serum Hsp70 on the ratio of CD3^−^/CD94^+^ NK cells, patients with squamous cell (*n* = 25) and adenocarcinoma (*n* = 18) were divided into groups with high (≥8.5% in squamous cell and ≥6.5% in adenocarcinoma) and low (<8.5% in squamous cell and <6.5% in adenocarcinoma) median percentages of CD3^−^/CD94^+^ NK cells. It appeared that patients with squamous cell NSCLC with a high CD3^−^/CD94^+^ NK cell ratio had significantly lower serum Hsp70 levels than the corresponding group with a low ratio of CD3^−^/CD94^+^ NK cell ratio (*p* = 0.048) (Figure 5A). The CD94^+^ NK cell population was also found to be positive for CD69, which indicates that these NK cells are active. Patients with high percentages of CD94^+^/CD69^+^ NK cells have low Hsp70 serum levels and also a lower GTV, which indicates that these NK cells might be able to control the growth of mHsp70-positive tumor cells. In contrast, patients with adenocarcinoma showed no significant differences with respect to the ratio of CD3^−^/CD94^+^ NK cells and serum Hsp70 levels (*p* = 0.908) (Figure 5B). These findings might indicate that squamous cell NSCLC is more immunogenic than adeno NSCLC.

## Discussion

Many lung tumors are diagnosed at advanced stages, which often restrict curative-intent treatment. In bronchial carcinoma, first diagnosis can be delayed by unspecific symptoms like coughing or dyspnea, which is also seen in inflammatory diseases of the lung, such as chronic obstructive pulmonary disease (COPD) or pneumonia. Apart from that, the majority of patients with the diagnosis COPD are smokers who additionally have an increased risk of developing lung cancer. Consequently, there is an urgent need for novel tumor biomarkers that can distinguish malignant from benign diseases. In contrast to normal cells, tumor cells frequently present Hsp70 on their surface. Membrane Hsp70-positive tumor cells have the capacity to actively secrete Hsp70 in lipid vesicles with molecular characteristics of exosomes (8, 9, 12). In a large variety of different malignant tumor entities, elevated Hsp70 levels in the serum could be detected (4, 5) which reflect a mHsp70-positive tumor phenotype. Herein, we could show significantly elevated levels of Hsp70 in the peripheral blood circulation of patients with squamous cell and adeno NSCLC when compared to healthy individuals. Previous work of our group has demonstrated that differences exist in Hsp70 serum levels in patients with inflammatory diseases, such as chronic hepatitis or liver cirrhosis and tumors, such as hepatocellular carcinoma (HCC) (25). All patients exhibited elevated Hsp70 levels in the serum compared to healthy controls, but the highest Hsp70 levels were detected in the group of tumor patients. In line with these findings, it has been demonstrated that NSCLC patients have higher Hsp70 levels in the blood than patients with COPD (26). These findings might provide a first hint that Hsp70 could have the potential as a tumor-specific biomarker, which is able to distinguish inflammatory and tumor diseases.

Since liposomal Hsp70, which can be quantified in the serum and plasma of patients, is derived from viable tumor cells (13, 27), we were interested to study the impact of RCT on serum Hsp70 levels in a small cohort of six NSCLC patients diagnosed with NSCLC stage IIIA and IIIB from whom blood was taken at diagnosis and after RCT. Despite a slight drop directly after completion of RCT, serum Hsp70 levels remained significantly higher than those in healthy individuals. This means that it might be possible to determine the Hsp70 tumor phenotype in the serum of patients not only at diagnosis but also during RCT.

Tumor staging in the follow-up period (2–3 months after RCT) revealed clinical responses, such as partial response or stable disease in these patients. Future studies on a larger patient cohort will elucidate whether clinical responses can be determined by a drop in the serum Hsp70 levels since the major part of circulating Hsp70 is actively released in a lipid-bound form by viable tumor cells (13). In order to further test this hypothesis, the GTV was compared to the serum Hsp70 levels. Herein, we could show that a small GTV was associated with low Hsp70 and a large GTV with high serum Hsp70 levels in NSCLC patients (*n* = 55; collective #2). Furthermore, a significant correlation between serum Hsp70 levels and PET-based GTV was shown using Spearman’s Rank Order Correlation. A potential correlation of the Hsp70 levels with the UICC stage has to be performed in a patient cohort with a more balanced distribution of different UICC stages. Studies of Zimmermann et al. (26) have shown that the Hsp27 and Hsp70 serum levels could discriminate clinical stages in NSCLC and the group of Bauer et al. (28) has shown that the tumoral expression of both HSPs might provide useful biomarkers for risk stratification of UICC stage I/II colon cancer. Considering the potential prognostic and predictive quality of tumor volume and its changes during RT of cancer (29, 30), serial GTV registrations at different time points before, during and after RT by CT, MRI, or PET will be determined together with serum Hsp70 levels in ongoing studies.

Nevertheless, further research is necessary to assess in more detail how homogeneously membrane Hsp70 is expressed in tumor cells within one tumor or in tumors of different patients in order to validate a direct correlation between serum Hsp70 levels and the viable tumor mass. Immunohistochemistry data reveal that tumor cells, but not the surrounding normal tissue, are Hsp70 positive. Equally important is to determine which factors can influence the active secretion of Hsp70-containing vesicles by tumor cells. In the tissue of patients with squamous cell carcinoma of the head and neck, a high membrane Hsp70 expression on viable tumor cells was found to be associated with high serum Hsp70 levels (31). Salamuta S. Mambula observed a re-binding of extracellular Hsp70 to the cell surface of prostate carcinoma cells after its release (32). This phenomenon might also have an impact on circulating levels of Hsp70.

Apart from the fact that high membrane Hsp70 expression levels are associated with and aggressive tumor phenotype, radioresistance (16), and tumor progression (33), Hsp70 can also provide a target for the innate immune system (11, 34, 35). In general, the immune system of each individual human blood donor is highly individual, depending on the genetic constitution combined with the exposition to various antigens during life. Patients with solid tumors often show an immunosuppressed immune phenotype due to a variety of tumor immune escape mechanisms (36). In the present study, we intended to detect differences in the immune phenotype of patients with NSCLC of different histology and healthy human individuals. Considering that patients with squamous cell and adenocarcinoma had significantly lower percentages of lymphocytes in the peripheral blood than healthy controls (Figure 4A), our findings confirm that the immune system is essential for tumor control. Since membrane Hsp70 acts as a recognition structure for Hsp70-peptide pre-activated NK cells (35), we asked the question whether lipid-bound, circulating Hsp70 has an impact on the immune phenotype of peripheral blood lymphocytes (PBL). Flow cytometric analysis of the blood of patients with squamous cell carcinoma showed decreased percentages of B cells but elevated percentages of activated NK cell subpopulations in patients with squamous cell, but not adeno NSCLC. Significantly increased percentages of CD69^+^/CD94^+^ NK cells were found in these patients compared to the healthy donors and adenocarcinoma patients. We could show that high serum Hsp70 levels are associated with a larger GTV in squamous cell but not adeno NSCLC. Regarding Figures 5A,B, patients with a lower percentage of CD94^+^/CD69^+^ activated NK cells have higher Hsp70 serum levels in squamous cell NSCLC. Since high Hsp70 serum levels are associated with a larger GTV we speculate that CD94^+^/CD69^+^ activated NK might be able to control growth of membrane Hsp70-positive tumor cells. Depending on its subcellular localization Hsp70 exerts dual functions. On the one hand, high intracellular and membrane-bound Hsp70 levels protect tumor cells from apoptotic cell death and thus mediate therapy resistance; on the other hand, membrane Hsp70 acts as a recognition structure for activated NK cells. Highly malignant tumor cells that secrete large amounts of Hsp70 might escape protective antitumor immunity by inducing tolerance, and therefore high Hsp70 levels that are associated with a larger GTV might be associated with a suppression of C-type lectin-positive NK cells. Vice versa, high percentages of CD94^+^/CD69^+^ NK cells can control growth of mHsp70-positive squamous cell carcinomas and thus serum levels of Hsp70 are lower. In case of adeno NSCLC, no correlation of the percentage of CD94^+^/CD69^+^ NK cells and serum Hsp70 levels were observed. This finding might be attributed to the fact that adenocarcinomas are less immunogenic.

Previously, it has been demonstrated that the cell surface density of the C-type lectin receptor CD94 was up-regulated on NK cells after stimulation with Hsp70-peptide TKD (aa_450–463_) and low-dose IL-2 (17). Apart from Hsp70, it is known that CD94 interacts with non-classical HLA-E molecules (37), and serves either as an activating or inhibitory receptor depending on the NKG2C or NKG2A co-receptor (38). In Hsp70 membrane-positive SCCHN patients, even 2 years after surgery and radiation therapy, the expression density of CD94 and NKG2D on NK cells was found to be significantly up-regulated (31). An increased expression of CD69 on NK cells is associated with an increased cytotoxic activity, proliferation, TNF-α production and the induction of further activation markers, such as CD25 and ICAM-1 (39). Our present data indicate that an increased percentage of CD69 and CD94 positive NK cells is only present in the blood of patients with squamous cell but not of adeno NSCLC patients and a significant association of the CD94 expression with serum Hsp70 could be also only detected in the group of squamous cell NSCLC patients (Figures 5A,B).

According to the diversity of their gene expression patterns, adenocarcinoma can be divided into subgroups with different outcome in overall survival (40). In squamous cell carcinoma of the lung, a reinforcement of the innate immune response by danger signals, such as circulating Hsp70, might be favorable. NK cells are not only able to detect “missing self” on malignant cells (41), but also can recognize membrane Hsp70 if expressed in a tumor-specific manner (42). Experimental mouse models indicate that the development of tumor-specific CD8^+^ cytotoxic T cell responses is highly dependent on the NK cell-mediated elimination of tumor cells (43, 44) through the secretion of IFN-γ. Also macrophages and dendritic cells are activated by IFN-γ and TNF. An 11-year follow-up epidemiologic survey has shown that the paucity of activated NK cells was associated with an increased risk to develop cancer (45). Taken together our data indicate that NK cells as the first line of defense might play a major role in the control of squamous cell NSCLC. The danger molecule Hsp70 in the presence of pro-inflammatory cytokines, such as IL-2, might support the immune system to reinforce immunity against cancer.

## Conclusion

We could show that Hsp70 detected by the lipHsp70 ELISA can serve as a tumor biomarker in liquid biopsies of patients with squamous cell and adeno NSCLC. Due to the fact that vesicular, lipid-bound Hsp70 predominantly originates from viable tumor cells, a correlation of serum Hsp70 levels with GTV was found. This finding is in accordance to the result that changes in tumor volume during radiotherapy in NSCLC patients have potential prognostic and predictive value (46, 47).

Compared to healthy individuals, NSCLC patients have decreased lymphocyte counts in general. However, a comparison of lymphocyte subpopulations in NSCLC patients with different histology revealed elevated percentages of CD69^+^/CD94^+^ NK cells in squamous cell but not adeno NSCLC patients. This might provide a hint that squamous cell NSCLC is more immunogenic than adeno NSCLC. High serum Hsp70 levels are associated with a larger GTV and lower percentage of CD69^+^/CD94^+^ NK cells. This might indicate that activated NK cells might be able to control growth of mHsp70-positive tumors in squamous cell NSCLC patients. If tumor escape mechanism suppress NK cell activation mHsp70-positive tumors cannot be killed.

## Author Contributions

SG and CO contributed equally to the study; SG, CO, DV, and GM conceived and designed the experiments and wrote the paper; SG, SS, HS, PM, MJ, and FP performed the experiments, and or analyzed the data; DV and SC did proof-reading of the paper and gave clinical advice.

## Conflict of Interest Statement

The authors declare that the research was conducted in the absence of any commercial or financial relationships that could be construed as a potential conflict of interest.
